# Therapeutic Effect and Underlying Mechanism of Blue Mussel (*Mytilus galloprovincialis*) Oil on Adjuvant-Induced Rheumatoid Arthritis in Rats

**DOI:** 10.3390/nu18020215

**Published:** 2026-01-09

**Authors:** Xin Yu, Xueyuan Fu, Fen Du, Chuyi Liu, Changwei Wang, Xiaomei Feng, Wanxiu Cao, Qingjuan Tang

**Affiliations:** 1 College of Food Science and Engineering, Ocean University of China, Qingdao 266100, China; yuxin_0415@126.com; 2 Marine Biomedical Research Institute of Qingdao, Ocean University of China, Qingdao 266100, China; fuxueyuan250@sina.com (X.F.); dufen0402@163.com (F.D.); liucy@ouc.edu.cn (C.L.); wchw209@126.com (C.W.); fengxmw@sina.com (X.F.); 3 Qingdao Marine Biomedical Research Institute Co., Ltd., Qingdao 266100, China

**Keywords:** blue mussel oil, rheumatoid arthritis, anti-inflammatory, lipid metabolites

## Abstract

Background: Rheumatoid arthritis (RA) is a chronic autoimmune disease characterized by synovitis. The prevalence of RA is estimated to be 0.5–1% worldwide. Methods: This work investigated the therapeutic effects and underlying mechanisms of blue mussel (*Mytilus galloprovincialis*) oil (BMO) on RA in rats, using green-lipped mussel oil (GMO) and Antarctic krill oil (KO) as controls. Results: The results suggested that BMO, GMO, and KO all alleviated paw swelling in rats and reduced serum levels of rheumatoid factor (RF), anti-cyclic citrullinated peptide (anti-CCP) antibody, and pro-inflammatory cytokines such as TNF-α and IL-17. Histopathological assessment further revealed that BMO, GMO, and KO prevented synovial fibroplasia, mitigated inflammatory cell infiltration, and improved cartilage damage in ankle joints. Overall, BMO exhibited slightly superior alleviating effects compared with GMO and KO. Plasma lipidomics analysis revealed that the lipid metabolites altered by BMO showed significant correlations with RA-related indicators, particularly pro-inflammatory cytokines. Functional enrichment analysis suggested the involvement of inflammation-related pathways, particularly the NF-κB signaling pathway. Further validation demonstrated that BMO effectively suppressed the production of inflammatory cytokines (TNF-α, IL-17) and the expression of NF-κB p65, JAK2, and STAT3 proteins in synovial tissue. And IL-17 production in footpad tissues is closely associated with CD3-positive T cells. Similar effects were also observed for GMO and KO. Conclusions: Collectively, BMO might ameliorate RA by inhibiting NF-κB and JAK2/STAT3 signaling pathways.

## 1. Introduction

Rheumatoid arthritis (RA) is a long-term autoimmune disease characterized by inflammatory pain in multiple joints, ultimately leading to cartilage destruction, joint deformity, and loss of joint function [1]. RA has a global prevalence of 0.5–1% and substantially compromises patients’ quality of life and work ability [2,3]. RA is recognized as a national health priority in a number of countries, warranting coordinated public health strategies [4,5]. Although the exact pathogenesis of RA remains unclear, numerous studies have demonstrated that its primary symptoms arise from synovial inflammation. While synovitis is a predominant early feature of RA, the long-term progression of the disease often culminates in cartilage damage, bone erosion, and, ultimately, permanent disability [6,7]. Within the synovial tissue, activated T lymphocytes, B lymphocytes, and macrophages contribute to progressive damage of the joints and cartilage [8,9]. These immune cells secrete a broad spectrum of pro-inflammatory cytokines and inflammatory mediators, which collectively drive extensive tissue injury and amplify secondary inflammatory responses during arthritis progression [10]. Additionally, by initiating autoimmune processes, T cells drive chronic synovitis and, ultimately, joint destruction [11].

Current clinical management of RA involves the use of multiple drug classes, such as disease-modifying anti-rheumatic drugs (DMARDs), nonsteroidal anti-inflammatory drugs (NSAIDs), glucocorticoids, and biologic agents. However, the efficacy of these drugs remains limited and is often accompanied by significant side effects [12], including gastrointestinal adverse reactions, cardiovascular damage, fractures and joint injuries, as well as comorbidities such as pneumonia and diabetes [13]. Biologics, such as monoclonal antibodies, are not only expensive and unsuitable for all patients, but may also increase the risk of infection [4]. Therefore, efforts to advance safe and effective drugs for RA treatment remain a major objective in this field of research.

Mussel oil (MO) is a lipid rich in ω-3 polyunsaturated fatty acids (PUFAs), such as docosahexaenoic acid (DHA) and eicosapentaenoic acid (EPA), the majority of which exist in phospholipid form [14]. Studies have shown that phospholipid-bound PUFAs exhibit superior bioavailability compared to triglyceride-bound forms [15]. It has been established that blue mussel lipid extract was more effective than fish oil in alleviating inflammation in diabetic rats, and MO demonstrated superior efficacy compared with fish oil in reducing the risk of atherosclerosis in mice [16,17]. The lipid composition of MO primarily comprises phospholipids (PLs), triglycerides (TGs), sterols (STs) and free fatty acids (FFAs) [18], with PLs accounting for over 50% of the total lipids [14], particularly phosphatidylcholine (PC), phosphatidylserine (PS) and phosphatidylethanolamine (PE) [19]. Furthermore, the mussel serves as an excellent source of plasmalogen [20]. Plasmalogen molecules contain characteristic vinyl ether bonds that confer anti-inflammatory, anti-oxidative stress, and anti-tumor properties.

Clinical studies have shown that Lyprinol®, a lipid extract derived from the New Zealand green-lipped mussel (GMO), can be a safe and effective adjunctive therapy for arthritis [21,22]. The hard-shelled mussel oil has been demonstrated to alleviate pathological symptoms in the ankle joints of arthritic rats [23]. Furthermore, it has been shown to improve clinical activity scores in RA patients [24]. A randomized crossover dietary intervention trial revealed that consumption of the blue mussel (*Mytilus galloprovincialis*) significantly reduced disease symptoms in female RA patients [25,26]. Furthermore, McPhee et al. reported the ameliorative effects of blue mussel oil (BMO) on adjuvant-induced RA in rats [27]. However, the inflammatory assessments in these study were limited to basic measures, such as body weight, joint swelling, and erythrocyte sedimentation rate. The anti-RA bioactive properties and underlying mechanisms of action of BMO remain to be fully elucidated.

This study aimed to systematically investigate the therapeutic efficacy of BMO in a rat RA model induced by complete Freund’s adjuvant (CFA) and to explore its potential mechanisms of action.

## 2. Materials and Methods

### 2.1. Ethical Approval

This study was approved by the Ethics Committee of Ocean University of China (OUC-AE-2024-030). All animal experimental procedures were conducted in accordance with the Guidelines for Care and Use of Laboratory Animals of Ocean University of China.

### 2.2. Primary Materials

BMO and GMO were extracted from blue mussels and green-lipped mussels, respectively, both purchased from a market in Qingdao, Shandong Province, China. BMO was prepared by our laboratory. The GMO was extracted by supercritical CO_2_ (China Harbin Essen Biotechnology Co., Ltd., Harbin, China). Antarctic krill oil (KO) was purchased from Hancheng Yuanbeibei Biotechnology Co., Ltd., Shanxi, China. The composition of fatty acids in BMO, GMO, and KO was presented in Table S1. All custom animal feed was obtained from XIAOSHUYOUTAI Biotechnology Co., Ltd., Beijing, China. (SCXK (Jing) 2023-0010).

### 2.3. BMO Extraction Method

Fresh blue mussels were deshelled and freeze-dried. The resulting freeze-dried powder was suspended in 10 volumes of ultrapure water and stirred thoroughly. The solution pH was carefully regulated to a value of 2.0, after which pepsin (0.10%) was added. Mixture was stirred, sealed, and enzymatically subjected to hydrolysis at 37 °C (3 h). Enzyme activity was terminated by heating at 90 °C for 10 min. The hydrolysate was centrifuged at 4000 rpm (20 min) to separate supernatant and precipitate, which were freeze-dried separately. Subsequently, the lyophilized supernatant and precipitate were each extracted with 10 volumes of anhydrous ethanol and subjected to ultrasonication at 400 W for 3 h. Upon completion of centrifugation at 10,000 rpm (10 min), supernatants were collected, concentrated, and blended to obtain BMO.

### 2.4. Gas Chromatography-Mass Spectrometry (GC-MS) Analysis

Fatty acid compositions of BMO, GMO, and KO were analyzed using GC-MS (Agilent 7890A-5975C, Agilent Technologies, Santa Clara, CA, USA). The system was fitted with an HP-5MS capillary column (30 m × 0.25 mm × 0.25 μm). Samples were derivatized by methylation, followed by extraction with n-hexane prior to GC-MS analysis [28]. An injection volume of 1 μL. The column oven was programmed to start at 50 °C, ramped to 200 °C and held for 1 min, and then further increased to 300 °C. Electron ionization (EI) was conducted at an energy level of 70 eV. Temperatures of ion source, injector, and auxiliary heater were maintained at 230 °C, 300 °C, and 280 °C, respectively. Mass spectral data were recorded across an *m*/*z* range of 40–800.

### 2.5. Animal Group and Medication Method

Fifty specific pathogen-free (SPF) grade sprague dawley (SD) male rats (approximately 300 g) were purchased from Jinan Pengyue Laboratory Animal Breeding Co., Ltd., Jinan, China (SCXK (Lu) 2022-0006). After one week of acclimatization, SD rats were randomly divided into five groups (n = 10), namely, Control group (Control), Model group (RA), Blue mussel oil group (RA + BMO), Green-lipped mussel oil group (RA + GMO), Antarctic krill oil group (RA + KO). The rats were maintained at 23 ± 2 °C. They were housed under a 12 h light-dark cycle.

**Figure 1:**
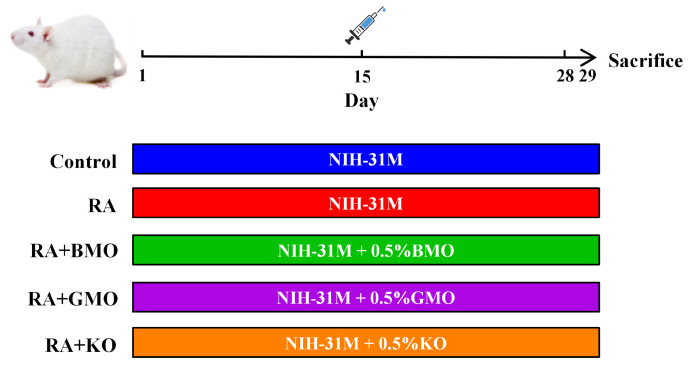
Schematic diagram of the animal experiment (n = 10). 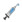: On day 15, the RA, RA + BMO, RA + GMO, and RA + KO groups were injected with CFA for modeling. Each group received a different diet starting on day 1.

The animal experimental design is illustrated in Figure 1. All the rats had free access to food and water. Different food were provided to each group from day 1 of the experiment. Control and RA groups were fed a standard NIH-31M diet throughout the experiment. The RA + BMO, RA + GMO, and RA + KO groups were fed NIH-31M diets supplemented with 0.5% BMO, 0.5% GMO, and 0.5% KO, respectively. The detailed compositions of the four diets are shown in Table S2. On day 15 of the study, rats in Control group received a subcutaneous injection of 0.1 mL of saline in the right hind paw. All other groups were injected with an equal volume of CFA (F5881, American Sigma-Aldrich, St. Louis, MO, USA) at the same site to establish a RA model.

### 2.6. Experimental Rat Sacrifice and Sample Collection

Upon completion of the study period, rats were rendered unconscious via intramuscular injection with 20% urethane (0.5 mL/100 g) (51-79-6, Sinopharm Chemical Reagent Co., Ltd., Shanghai, China). Blood was collected from abdominal aorta into Ethylene Diamine Tetraacetic Acid (EDTA) anticoagulant tubes as well as non-anticoagulated collection tubes.After centrifugation at 3000 rpm for 10 min at 4 °C, supernatants were collected and stored at −80 °C. Muscle tissues from right hind paw pads and intact ankle joints of rats were excised. Tissues immediately fixed in 4% paraformaldehyde solution (G1101, Wuhan Servicebio Technology Co., Ltd., Wuhan, China).

### 2.7. Determination of Paw Thickness and Arthritis Score

Thickness of right hind paw was measured every other day using a vernier caliper from 15 to 28 days. At study completion, arthritis scores were assessed based on the following criteria: severity of swelling (0 indicated no redness or swelling in the joints and toes, 0.5 indicated mild but definite joint swelling, 1 indicated severe redness and intense swelling) and the number of swollen toes (0.1 point per swollen toe). The maximum arthritis score was capped at 1.5.

### 2.8. Determination of Serum Biochemical Parameters and Inflammatory Factors

According to the ELISA kit (Qingdao Zhongxi Biotechnology Co., Ltd., Qingdao, China) instructions, serum concentrations of rheumatoid factor (RF), anti-cyclic citrullinated peptide (anti-CCP) antibody, TNF-α, IL-1β, IL-6, and IL-17 were measured.

### 2.9. Histomorphological Detection

The ankle joint tissues were decalcified in 10% EDTA at 4 °C for 30 days. Ankle joints and paw pad muscle tissues were dehydrated in a graded ethanol series (75%, 85%, 90%, and 95%), cleared twice in xylene. Then embedded in paraffin. Subsequently, the samples were cut into sections about 4 μm in thicknes. Hematoxylin and eosin (H&E), Masson’s trichrome (MT) and safranin O-fast green (safranin O) staining were conducted according to standard procedures [29,30]. Histopathological changes were assessed by light microscopy (Olympus, Tokyo, Japan).

### 2.10. Immunofluorescence (IF) and Immunohistochemical (IHC) Analysis

IF and IHC were performed on ankle joint sections as previously described [31]. Sections were respectively treated with TNF-α antibody (Ab6671, 1:500), IL-17 antibody (Ab79056, 1:500), NF-κB p65 antibody (Ab16502, 1:1000), JAK2 antibody (Ab108596, 1:500), STAT3 antibody (Ab68153, 1:500), and CD3 antibody (Ab11089, 1:200). All the antibodies were acquired from Abcam Company (Waltham, MA, USA). The Positive areas were quantified using ImageJ software (version 1.54g, National Institutes of Health, Bethesda, MD, USA).

#### 2.10.1. IF Analysis

Tissue sections were deparaffinized and rehydrated, followed by antigen retrieval in EDTA buffer (pH 8.0). After PBS washing, autofluorescence was quenched and nonspecific binding was blocked with BSA. The sections were incubated with primary antibodies overnight at 4 °C and then with fluorescently labeled secondary antibodies in the dark. The sections were mounted with antifade medium and stored at 4 °C.

#### 2.10.2. IHC Analysis

Tissue sections were deparaffinized and subjected to antigen retrieval in citrate buffer (pH 6.0). Endogenous peroxidase activity was blocked with 3% hydrogen peroxide, followed by blocking with 3% bovine serum albumin. The sections were incubated with primary antibodies overnight at 4 °C and then with secondary antibodies at room temperature. After counterstaining with hematoxylin, the sections were mounted and examined under a light microscope.

### 2.11. Ultra-Performance Liquid Chromatography-Mass Spectrometry (UPLC-MS) Analysis of Plasma Lipidomics

Each plasma sample (90 μL) was mixed with 270 μL of chloroform-methanol (2:1, *v/v*) to extract lipids. A mixed internal standard solution (4 μg/mL in methanol) was added to the sample. The mixture was subjected to ultrasonic extraction for 10 min, followed by incubation at −20 °C for 30 min. The mixture was centrifuged at 13,000 rpm and 4 °C for 10 min. Subsequently, 135 μL of the lower chloroform phase was collected and evaporated to dryness. The lipid residue was reconstituted in 180 μL of methanol-isopropanol solution (1:1, *v/v*), followed by sonication and centrifugation. Subsequently, 120 μL of the supernatant was transferred to an injection vial for UPLC-MS analysis.

UPLC was equipped with an ACQUITY UPLC BEH C8 column (1.7 μm, 100 mm × 2.1 mm, Waters). The column oven temperature was set at 55 °C, injection volume at 3 μL, and flow rate at 0.26 mL/min. Mobile phase A consisted of acetonitrile and water (6:4, *v/v*), containing 10 mM ammonium acetate. Mobile phase B was isopropanol and acetonitrile (9:1, *v/v*), containing 10 mM ammonium acetate. The elution program was as follows: 0–1.5 min, 32% B; 1.5–15.5 min, 32–85% B; 15.5–15.6 min, 85–97% B; 15.6–18.0 min, 97% B; 18.0–18.1 min, 97–32% B; 18.1–20.0 min, 32% B. The MS was carried out in positive and negative ion mode, with a mass range of 150–1500 *m*/*z*, a capillary temperature at 300 °C, a aux gas heater temperature at 350 °C, a sheath gas flow rate at 45 Arb, and a aux gas flow rate at 10 Arb.

### 2.12. Bioinformatics Analysis

Differentially expressed lipid metabolites were identified based on a *p*-value < 0.05 and a fold change (FC) ≥ 1.2 or ≤1/1.2. Functional enrichment and KEGG pathway analyses were subsequently conducted using the KEGG database (https://www.genome.jp/kegg/, accessed on 23 January 2025) to identify significantly enriched metabolic pathways.

### 2.13. Statistical Analysis

The data are expressed as mean ± standard deviation (SD). Statistical analysis was conducted using SPSS 22, with one-way ANOVA followed by LSD post-hoc test for intergroup comparisons. A *p*-value < 0.05 was considered statistically significant. Data visualization was performed using GraphPad Prism 8.

## 3. Result

### 3.1. Paw Thickness and Arthritis Score

**Figure 2:**
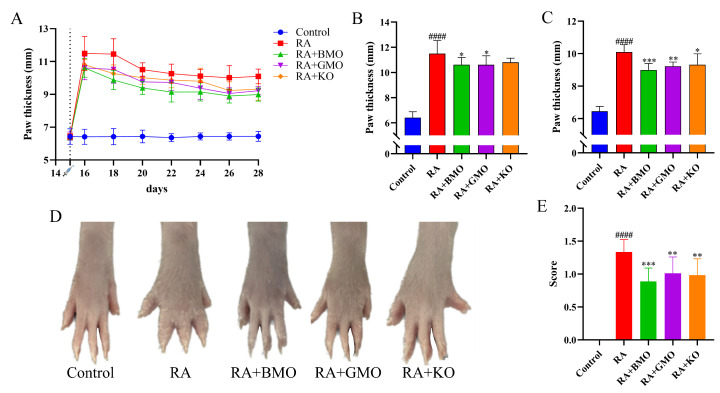
Effect of BMO on arthritis index score of RA rats (n = 10). (**A**) Paw thickness change during modeling. (**B**) Paw thickness on Day 16. (**C**) Paw thickness on Day 28. (**D**) Paw swelling degree on Day 28. (**E**) Arthritis index score on Day 28. ^####^ *p* < 0.0001, compared with the Control group. * *p* < 0.05, ** *p* < 0.01, *** *p* < 0.001, compared with the RA group. 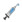: On day 15, Control group was injected with normal saline; RA, RA + BMO, RA + GMO, and RA + KO groups were injected with CFA for modeling.

Joint swelling and deformation are among the clinical symptoms of RA. Following CFA injection, paw thickness in the RA + BMO, RA + GMO, and RA + KO groups remained consistently lower than that in the RA group (Figure 2A). Compared with the Control group, the RA group exhibited a significant increase in paw thickness on day 16 (*p* < 0.0001). BMO and GMO caused a significant reduction in paw thickness compared to the RA group on day 16 (Figure 2B, *p* < 0.05). The paw thickness in the RA group was significantly higher than that in the Control group on day 28 (*p* < 0.0001, Figure 2C). The RA + BMO, RA + GMO, and RA + KO groups exhibited significantly lower paw thickness than the RA group on day 28 (*p* < 0.05). As illustrated in Figure 2D,E, the RA group displayed the most severe swelling in the right hind paw. In contrast, BMO, GMO and KO exhibited marked improvement in joint swelling (*p* < 0.01). However, on days 16 and 28, the paw thickness in the BMO, GMO, and KO groups remained significantly higher than that in the Control group (Figure 2B,C). These results indicated that although BMO effectively alleviated CFA-induced RA symptoms, it was not sufficient to fully reverse the RA-induced damage.

### 3.2. Serum Biochemical Parameters and Inflammatory Factors

#### 3.2.1. Diagnostic Markers of Rheumatoid Arthritis

**Figure 3:**
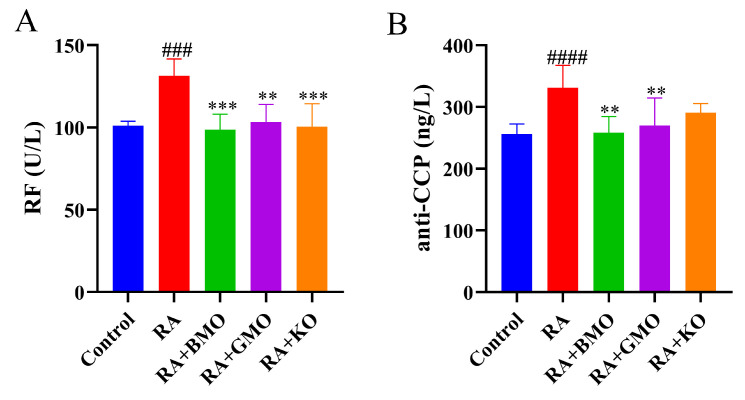
Effect of BMO on diagnostic factors in serum of RA rats (n = 10). (**A**) Rrheumatoid factors (RF). (**B**) Anti-cyclic citrulline peptide (anti-CPP) antibody. ^###^ *p* < 0.001, ^####^ *p* < 0.0001, compared with the Control group. ** *p* < 0.01, *** *p* < 0.001, compared with the RA group.

RF and anti-CCP serve as important serological indicators for the diagnosis of RA. As shown in Figure 3, both RF and anti-CCP levels in the RA group were markedly higher than those in the Control group (*p* < 0.001). Relative to the RA group, BMO and GMO interventions significantly reduced serum levels of both RF and anti-CCP (*p* < 0.01). KO intervention also resulted in a pronounced decrease in RF concentration (*p* < 0.01). These results suggested that, similar to GMO and KO, BMO may effectively alleviated RA symptoms.

#### 3.2.2. Inflammatory Factors

**Figure 4:**
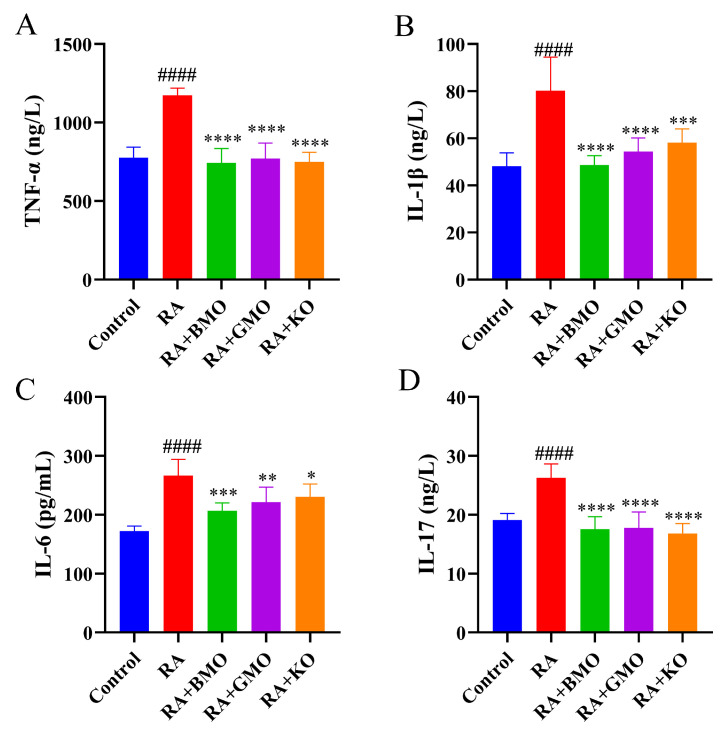
Effect of BMO on inflammatory factors in serum of RA rats (n = 10). (**A**) TNF-α; (**B**) IL-1β; (**C**) IL-6; (**D**) IL-17. ^####^ *p* < 0.0001, compared with the Control group; * *p* < 0.05, ** *p* < 0.01, *** *p* < 0.001, **** *p* < 0.0001, compared with the RA group.

Pro-inflammatory cytokines play crucial roles in the development of RA, synovial inflammation, and the occurrence of bone destruction. Compared with Control group, serum levels of TNF-α, IL-1β, IL-6, and IL-17 were markedly elevated in rats with RA (*p* < 0.0001, Figure 4). In contrast, levels of these cytokines in RA + BMO, RA + GMO, and RA + KO groups were markedly reduced compared with RA group (*p* < 0.05, Figure 4). Notably, BMO supplementation restored serum levels of TNF-α, IL-1β, and IL-17 in RA rats to normal levels.

### 3.3. Pathological Analysis

**Figure 5:**
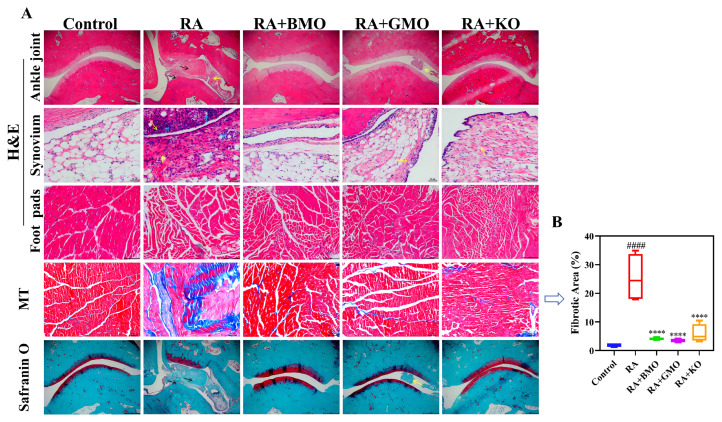
Effect of BMO on the morphology of ankle joint and synovium of rats (n = 10). (**A**) Representative H&E, MT and Safranin O results. (**B**) Fibrotic area in MT. Scale bar: 50 μm (synovium H&E and MT), 200 μm (other sections). Yellow arrows indicate severe synovial fibrosis; blue arrows indicate inflammatory cell infiltration; black arrows indicate cartilage destruction and bone erosion. ^####^ *p* < 0.0001, compared with the Control group. **** *p* < 0.0001, compared with the RA group.

We performed histopathological examination of ankle joints to further investigate pathological changes. Figure 5 illustrates that rats in Control group exhibited normal histological structure, such as smooth articular surfaces, normal joint space and synovial structure, along with intact cartilage and bone tissue. In contrast, rats in the RA group showed uneven articular surface, severe synovial fibrosis (yellow arrows), extensive inflammatory cell infiltration (blue arrows), pannus formation, and disorganized and fibrotic muscle tissue in footpads. Additionally, safranin O staining revealed severe joint damage and bone destruction in RA group. Compared with RA group, RA + BMO, RA + GMO, and RA KO groups significantly improved synovial fibrosis (yellow arrows), cartilage destruction and bone erosion (black arrows). Thus, BMO, GMO, and KO all mitigated pathological changes in the ankle joints and synovial tissue of RA rats, demonstrating protective effects against CFA-induced joint damage.

### 3.4. Plasma Lipid Metabolite Analysis

#### 3.4.1. Screening of Significantly Differential Lipid Metabolites

**Figure 6:**
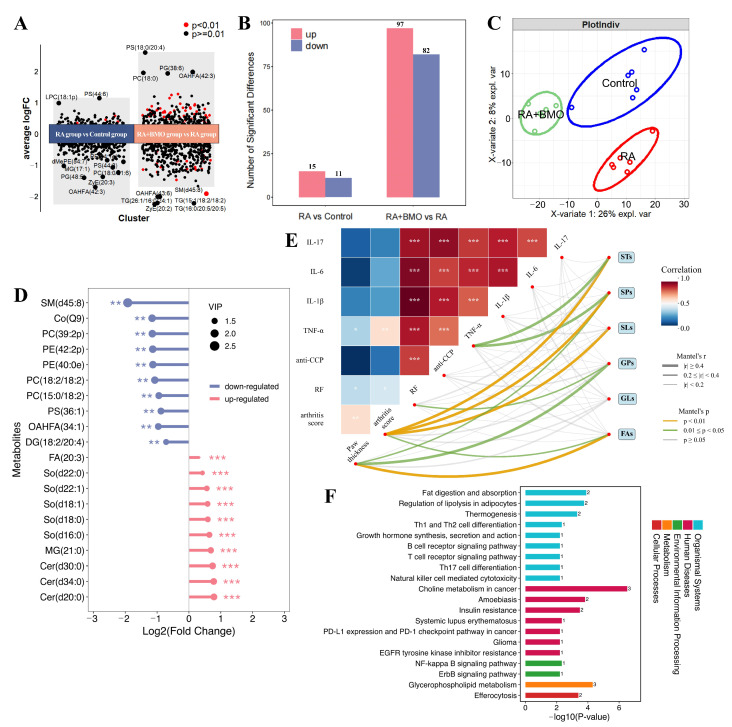
Effect of BMO on differential lipid metabolites in plasma of RA rats (n = 6). (**A**) Partial least squares-discriminant analysis (PLS-DA). (**B**) Statistical quantity of differential metabolites. (**C**) Volcano plot of differential metabolites. (**D**) Top 20 significant differential metabolites (RA + BMO vs. RA). (**E**) Mantel test of relationships between RA-related indexes and significant differential metabolites from lipid categories, including fatty acyls (FAs), glycerolipids (GLs), glycerophospholipids (GPs), saccharolipids (SLs), sphingolipids (SPs), and sterol lipids (STs). (**F**) Significant differential KEGG pathways (RA + BMO vs. RA). * *p* < 0.05, ** *p* < 0.01, *** *p* < 0.001.

Lipid metabolism dysregulation may represent an important characteristic of RA pathogenesis. In this study, targeted lipidomic analysis was performed, which provides quantitation of 919 individual lipid species from 25 lipid classes. Partial least squares-discriminant analysis (PLS-DA) analysis revealed that the overall distribution patterns of lipid metabolites differed significantly among the groups (Figure 6A). A total of 26 and 179 differential metabolites (*p* < 0.05) were identified between RA group and Control group and between RA + BMO group and RA group, respectively (Figure 6B). The volcano plot of top 10 metabolites ranked by fold change is presented in Figure 6C. It illustrates the distribution of 97 increased and 82 decreased lipid species between RA + BMO group and RA group. The top 10 differential metabolites between the RA and Control groups consisted primarily of glycerophospholipids (GPs), such as PS, PE, lysophosphatidylcholine (LPC). In contrast, those between the RA + BMO and RA groups were mainly PS, PC, phosphatidylglycerol (PG), TG, and sphingomyelin (SM). The results suggested that BMO intervention significantly regulated the plasma metabolome of RA rats.

#### 3.4.2. Classification of Significantly Differential Lipid Metabolites

As shown in Figure S1, we classified the intergroup differential lipid metabolites. Specifically, the significantly altered metabolites between the RA and Control groups were predominantly GPs. In contrast, the RA+BMO group versus RA group comparison revealed more diverse differential lipid metabolites, including GPs, sphingolipids (SPs) and glycerolipids (GLs).

Then top 20 significantly differential metabolites (*p* < 0.05, fold change > 1) are depicted in Figure S2 and Figure 6D. Compared with Control, RA group exhibited markedly elevated levels of LPC species in serum. Compared to RA, RA+BMO group demonstrated a significant increase in several SPs (such as sphingoshine (So), ceramides (Cer)). Moreover, Mantel test revealed that multiple GPs and SPs were markedly associated (*p* < 0.05) with RA-related indicators (Figure 6E). These results were confirmed in analyses of individual lipid species (Figure S3). For instance, PS (36:1) showed a strong positive correlation with serum TNF-α (r > 0.6, *p* < 0.05).

#### 3.4.3. Functional Enrichment Analysis of Significantly Differential Lipid Metabolites

Figure S4 and Figure 6F presented the Level 3 Kyoto Encyclopedia of Genes and Genomes (KEGG) pathway distribution of differential metabolites. Compared to Control, RA group exhibited significant reduction in fatty acid metabolism, indicated by the down-regulation of linoleic acid metabolism, alpha-linolonic acid metabolilsm, glycerophospholipid metabolism, and arachidonic acid metabolism (*p*-value < 0.05). In the comparison between the RA + BMO group and RA group, the pathways significantly down-regulated within lipid metabolism included fat digestion and absorption, regulation of lipolysis in adipocytes, and glycerophospholipid metabolism (*p*-value < 0.05). Notably, BMO intervention also remarkably suppressed inflammation-related pathways, particularly Th17 cell differentiation and NF-κB signaling pathway (*p*-value < 0.05).

### 3.5. Analysis of TNF-α, IL-17 and CD3

**Figure 7:**
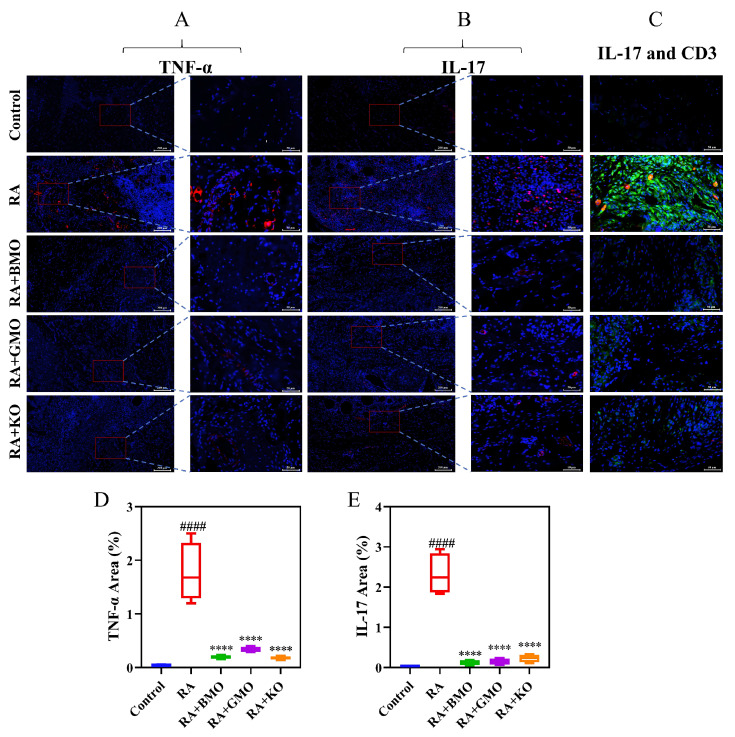
Effect of BMO on the expression levels of TNF-α, IL-17 and CD3. (n = 6). Representative merged IF images (scale bar: 200 μm) and enlarged views (scale bar: 50 μm) showing (**A**) TNF-α and (**B**) IL-17 expression in the synovium of rat ankle joints. (**C**) Co-localization of IL-17 and CD3 in footpad tissues (scale bar: 50 μm): yellow signals indicate co-localized expression. The area of (**D**) TNF-α and (**E**) IL-17 positive expression. ^####^ *p* < 0.0001, compared with the Control group. **** *p* < 0.0001, compared with the RA group.

To further investigate the mechanism underlying the anti-RA activity of BMO, we detected the expression levels of TNF-α and IL-17 in rat ankle joint tissues, as well as localization of IL-17 and CD3 in footpad tissues, by IF staining (Figure 7). Relative to Control group, RA group exhibited marked upregulation of both TNF-α and IL-17. In contrast, compared with RA group, the RA + BMO, RA + GMO, and RA + KO groups showed pronounced downregulation of TNF-α and IL-17 expression. Figure 7C showed that IL-17 signals were markedly increased in the footpad tissues of RA rats and were largely co-localized with CD3-positive cells. In contrast, IL-17 immunoreactivity and its co-localization with CD3-positive cells were reduced in the RA + BMO, RA + GMO, and RA + KO groups. These findings demonstrated that, akin to GMO and KO, BMO had effectively suppressed the expression of pro-inflammatory factors in the ankle joint tissues of RA rats. Moreover, the co-localization analysis suggested that IL-17 production is closely associated with CD3-positive T cells in RA footpad tissues, and that BMO treatment may attenuate T cell–related IL-17–mediated inflammatory responses.

### 3.6. Analysis of NF-κB p65, JAK2 and STAT3 in Rat Ankle Joints

**Figure 8:**
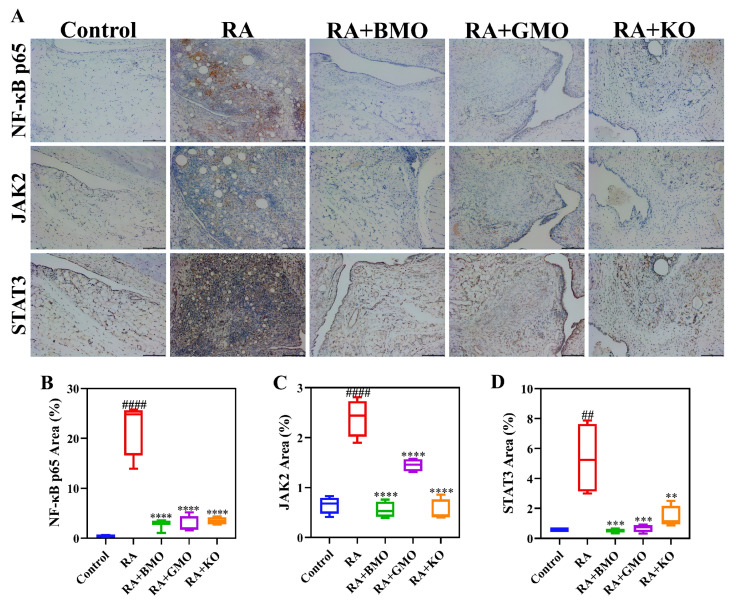
Effect of BMO on the expression levels of selected pathways associated with inflammation in ankle joint of rats (n = 6). (**A**) Representative IHC results (scale bar 200 μm): the brown areas indicate positive protein expression. The area of (**B**) NF-κB p65, (**C**) JAK2 and (**D**) STAT3 positive expression. STAT3 represents total STAT3 protein. ^##^ *p* < 0.01, ^####^ *p* < 0.0001, compared with the Control group. ** *p* < 0.01, *** *p* < 0.001, **** *p* < 0.0001, compared with the RA group.

Based on the KEGG enrichment results, we investigated the expression of NF-κB and JAK2/STAT3 signaling pathways (Figure 8). The findings revealed that the expression of NF-κB p65, JAK2, and STAT3 within rat synovial tissues were markedly elevated in RA group when compared with Control group (*p* < 0.01). On the other hand, the significant decrease in the expression levels of these three proteins in synovial tissue occurred in RA + BMO group, RA + GMO group, and RA + KO group, respectively (*p* < 0.01). These results demonstrated that BMO, GMO, and KO might ameliorate CFA-induced ankle joint inflammation and synovial tissue hyperplasia in RA rats by suppressing both the NF-κB and JAK2/STAT3 signaling pathways.

## 4. Discussion

Consistent with the effects of GMO and KO, BMO treatment significantly alleviated RA symptoms in rats, as evidenced by reduced paw swelling and arthritis scores. In addition, BMO downregulated the serum levels of RA markers (RF and anti-CCP) and key pro-inflammatory cytokines (TNF-α, IL-1β, IL-6, and IL-17). Furthermore, BMO intervention markedly ameliorated joint and tissue damage by suppressing synovial fibroplasia, reducing inflammatory cell infiltration, and alleviating destruction of bone and cartilage, alongside improving structural damage in footpad muscle tissues. Our study further revealed that BMO remarkably modulated RA-associated lipid metabolites and effectively downregulated multiple inflammation-related pathways. Accordingly, BMO supplementation led to a significant reduction in the expression of TNF-α, IL-17, NF-κB p65, JAK2, and STAT3 proteins in rat ankle joints.

New Zealand GMO has been proven to alleviate symptoms of osteoarthritis and RA, demonstrating efficacy in relieving joint pain and promoting joint cartilage regeneration [32]. Similarly, KO has shown excellent anti-inflammatory properties, with clinical studies confirming its efficacy in improving osteoarthritis-related knee pain in adults aged 40 to 65 [33]. Therefore, GMO and KO were used as positive controls in the current study.

Joint swelling severity is a critical metric for evaluating anti-RA efficacy. In this study, BMO intervention effectively alleviated paw edema in RA rats and reduced arthritis scores. These findings are consistent with the previous report by Fu et al. regarding Greenshell mussel oil [24]. McPhee et al. isolated triacylglycerols from BMO and demonstrated that their intervention resulted in a 65% reduction in hind-paw swelling in RA rats [27]. Serum RF and anti-CCP levels were positively correlated with synovial hyperplasia and cartilage erosion in the joint cavity of RA patients [34]. In the present study, BMO treatment noticeably decreased serum RF and anti-CCP concentrations, suggesting that dietary BMO intake might attenuate articular lesion severity in RA rats. However, although paw thickness was reduced compared with the RA group, it remained significantly higher than that in the control group, indicating that BMO could not completely reverse RA-induced damage.

Pro-inflammatory cytokines, including TNF-α, IL-1β, IL-6, and IL-17, play pivotal roles in RA progression and are primary drivers of bone destruction. TNF-α potentiates secretion of IL-1β and IL-6, promoting inflammatory cell migration [35]. Notably, IL-1β is associated with cartilage degradation, inflammatory cell infiltration, and induction of IL-6 secretion [36]. Moreover, TNF-α and IL-1β synergistically stimulate osteoclast differentiation [37]. IL-6 and IL-17 contribute to synovial hyperplasia and inflammation in RA and can directly trigger cartilage erosion [38]. BMO effectively downregulated the secretion of these pro-inflammatory cytokines, which demonstrated potent suppression of the inflammatory response. This resulted in the exertion of anti-arthritic effects. However, how BMO modulates inflammatory cell expression has yet to be fully clarified and warrants further investigation.

Key pathological features of RA include pannus formation, proliferation, and synovial inflammation [39]. Histopathological examination showed that, like GMO and KO, BMO intervention markedly reduced paw swelling in RA rats. The synovial tissue exhibited no inflammatory hyperplasia, with preserved smooth surfaces in both articular cartilage and subchondral bone. Consistent with these findings, some researchers also demonstrated through histopathological analysis that intake of GMO effectively alleviated cartilage erosion in osteoarthritis rats [40]. Therefore, based on assessments of paw thickness, arthritis scores, serum biomarkers, and histopathological changes, it can be concluded that BMO exhibits significant anti-RA activity. Fatty acids can modulate inflammatory responses [41]. In the present study, BMO, GMO, and KO all exhibited anti-inflammatory effects in alleviating joint inflammation. Overall, BMO showed slightly superior efficacy compared with GMO and KO, which may be attributable to differences in their fatty acid compositions.

Lipidomics plays a critical role in elucidating the pathogenesis of RA. Through the screening of RA-specific biomarkers, lipidomics can identify potential therapeutic targets. Previous studies have demonstrated alterations in the lipid composition of plasma in RA patients, particularly in GPs (e.g., LPC, LPE, PC, PE, PS, PG, PI), and sphingolipids (e.g., So, SM) [42,43,44]. Lipidomics analysis revealed that the intake of BMO significantly modulated the levels of metabolites in GPs, SPs, and GLs metabolism. Notably, correlation analysis revealed significant associations between differential GPs metabolites (e.g., PC, PE, PS, PI) and serum pro-inflammatory cytokine levels. These metabolites are potential therapeutic targets for RA, but the precise mechanisms require further investigation.

Functional enrichment analysis revealed that BMO intervention inhibited inflammatory signaling pathways, including Th17 cell differentiation and NF-κB signaling pathway. Th17 cells secrete IL-17, which stimulates pro-inflammatory cytokine (e.g., TNF-α) production and leads to abnormal synovial hyperplasia. IL-17 exacerbates inflammatory responses and promotes synovial tissue remodeling in RA [45]. STAT3 protein plays a critical role in regulating Th17 cell differentiation, and IL-17 production is closely associated with STAT3 phosphorylation [46]. Furthermore, cytokines such as TNF-α activate the NF-κB pathway, thereby promoting the proliferation and differentiation of inflammatory cells in RA patients, contributing to synovial inflammation and subsequent joint damage [47]. The NF-κB and JAK2/STAT3 signaling pathways are key contributors to the development and progression of RA. Inhibiting these pathways reduces synovial hyperplasia in RA rats, which alleviates inflammatory progression in joint tissues [13]. Additionally, IL-17 enhances TNF-α-induced NF-κB activation, while TNF-α amplifies IL-17-driven STAT3 signaling, forming an inflammatory amplification loop [45,46,47]. Treatment with BMO considerably reduced the expression levels of TNF-α and IL-17 in the rat ankle joints. In addition, IL-17 production in the footpad tissues appears to be closely associated with CD3-positive T cells. Concurrently, the expression of key proteins in the NF-κB signaling pathway, including NF-κB p65, was also decreased. Therefore, these findings suggest that BMO reduced the expression of TNF-α and IL-17, inhibited the NF-κB signaling pathway, and noticeably alleviated inflammatory responses in the ankle joints of RA rats.

Our results showed that JAK2 and STAT3 protein expression was significantly elevated in RA rats, and BMO treatment effectively reduced the levels of both proteins. While the observed changes in total protein expression do not definitively indicate suppression of JAK2/STAT3 pathway activation, the marked decrease in the upstream cytokine IL-6 implies a potential inhibitory effect of BMO on this signaling pathway. Additional investigations, including assessment of JAK2 and STAT3 phosphorylation and downstream target genes, are needed to further substantiate this hypothesis.

## 5. Conclusions

In conclusion, BMO protects against joint and synovial damage in RA by modulating lipid metabolism and suppressing the NF-κB signaling pathway, which attenuates inflammation in the ankle joints. Furthermore, these findings provide preliminary evidence that BMO may modulate the JAK2/STAT3 signaling pathway in RA, contributing to its anti-inflammatory effects.

## Data Availability

Data generated and analyzed in this study can be obtained from the corresponding authors upon reasonable request.

## Supplementary Materials

The following supporting information can be downloaded at https://www.mdpi.com/article/10.3390/nu18020215/s1. Table S1: Fatty acids composition (% of total fatty acids) of BMO, GMO and KO; Table S2: Detailed compositions of the four diets; Figure S1: Effect of blue mussel oil on classfication of differential lipid metabolites in plasma of RA rats (n = 6). (A) RA group vs. Control group, (B) RA+BMO group vs. RA group. Differential lipid metabolites were identified based on *p* < 0.05 and |fold change| ≥ 1.2. FA: Fatty acyls; GL: Glycerolipids; GP: Glycerophospholipids; SL: Saccharolipids; SP: Sphingolipids; ST: Sterol Lipids; Figure S2: Top 20 significant differential lipid metabolites (RA group vs. Control group) in plasma of RA rats (n = 6). The top 10 upregulated and top 10 downregulated metabolites with the smallest *p* values were selected. Red and blue dots indicate upregulated and downregulated metabolites, respectively. * *p* < 0.05; ** *p* < 0.01. Dot size reflects the VIP score; Figure S3: Correlation analysis between significant differential metabolites and RA related indexes (n = 6). Correlations were calculated using Spearman’s correlation analysis. Blue colors indicate positive correlations; red colors indicate negative correlations. *p* < 0.05 was considered statistically significant; Figure S4: Significant differential KEGG pathways (RA group vs. Control group) in plasma of RA rats (n = 6). Numbers on the bars indicate the number of differential metabolites mapped to each pathway, and bar colors denote different KEGG Level 1 categories.
