# Supplementary material for: Therapeutic Effect and Underlying Mechanism of Blue Mussel (Mytilus galloprovincialis) Oil on Adjuvant-Induced Rheumatoid Arthritis in Rats
Source: Nutrients. 2026 Jan 9;18(2):215. doi: 10.3390/nu18020215 (PMC12845126; doi:10.3390/nu18020215)
Supplement: Supplementary file 1 [file nutrients-18-00215-s001.zip › nutrients-4021653-supplementary.pdf]

Table S1. Fatty acids composition (% of total fatty acids) of BMO, GMO and KO.

Variables	BMO (%)	GMO (%)	KO (%)
C12:0	ND	ND	0.05
C14:0	7.68	9.83	1.00
C15:0	0.77	1.18	0.12
C16:0	ND	ND	29.67
C18:0	3.84	4.61	1.57
C20:0	0.38	ND	0.45
C22:0	ND	ND	0.12
C14:1n-5	0.17	ND	ND
C16:1n-7	23.87	17.61	26.96
C17:1n-7	0.13	ND	0.23
C18:1n-9	10.26	9.39	33.80
C20:1n-9	4.28	3.19	ND
C20:2n-9	1.31	1.18	ND
C18:2n-3	ND	0.53	ND
C18:3n-3(ALA)	1.19	1.21	ND
C18:4n-3	5.57	3.86	ND
C20:5n-3(EPA)	22.71	23.66	ND
C22:5n-3(DPA)	0.78	2.02	ND
C22:6n-3(DHA)	9.78	16.86	ND
C18:2n-6	5.10	2.51	6.04
C20:2n-6	0.82	0.42	ND
C20:3n-6	0.25	ND	ND
C20:4n-6	0.90	1.29	ND
C22:4n-6	0.20	0.65	ND
Total SFA	12.67	15.62	32.97
Total USFA	87.33	84.38	67.03

The content of fatty acids methyl esters of BMO, GMO, and KO were 73.06 mg/g, 71.53 mg/g, and 73.67 mg/g, respectively. ALA, Alpha linolenic acid; EPA, eicosapentaenoic acid; DPA, docosapentaenoic acid; DHA, docosahexaenoic acid; SFA, saturated fatty acid; USFA, unsaturated fatty acid; BMO, blue mussel oil; GMO, green-lipped mussel; KO, Antarctic krill oil; ND, not detected or negligible.

Table S2. Detailed compositions of the four diets.

Ingredient	NIH-31M (%)	NIH-31M+0.5% BMO (%)	NIH-31M+0.5% GMO (%)	NIH-31M+0.5% KO (%)
Ground whole wheat	35.17	35.17	35.17	35.17
Ground whole yellow corn	20.00	20.00	20.00	20.00
Ground whole oats	10.00	10.00	10.00	10.00
Wheat middlings	10.00	10.00	10.00	10.00
Fish meal (60% protein)	9.00	9.00	9.00	9.00
Soybean meal (47.5% protein)	5.00	5.00	5.00	5.00
Soy bean oil (no additives)	2.50	2.00	2.00	2.00
Alfalfa meal (17% protein)	2.00	2.00	2.00	2.00
Corn gluten meal (60% protein)	2.00	2.00	2.00	2.00
Dicalcium phosphate	1.50	1.50	1.50	1.50
Brewer's dried yeast	1.00	1.00	1.00	1.00
Ground limestone	0.50	0.50	0.50	0.50
Salt	0.50	0.50	0.50	0.50
NIH #31 vitamin premix	0.25	0.25	0.25	0.25
NIH #31 mineral premix	0.25	0.25	0.25	0.25
Choline chloride	0.13	0.13	0.13	0.13
L-lysine	0.10	0.10	0.10	0.10
DL-methionine	0.10	0.10	0.10	0.10
BMO	-	0.50	-	-
GMO	-	-	0.50	-
KO	-	-	-	0.50

BMO, blue mussel oil; GMO, green-lipped mussel; KO, Antarctic krill oil; "-" indicates that this ingredient was not added.

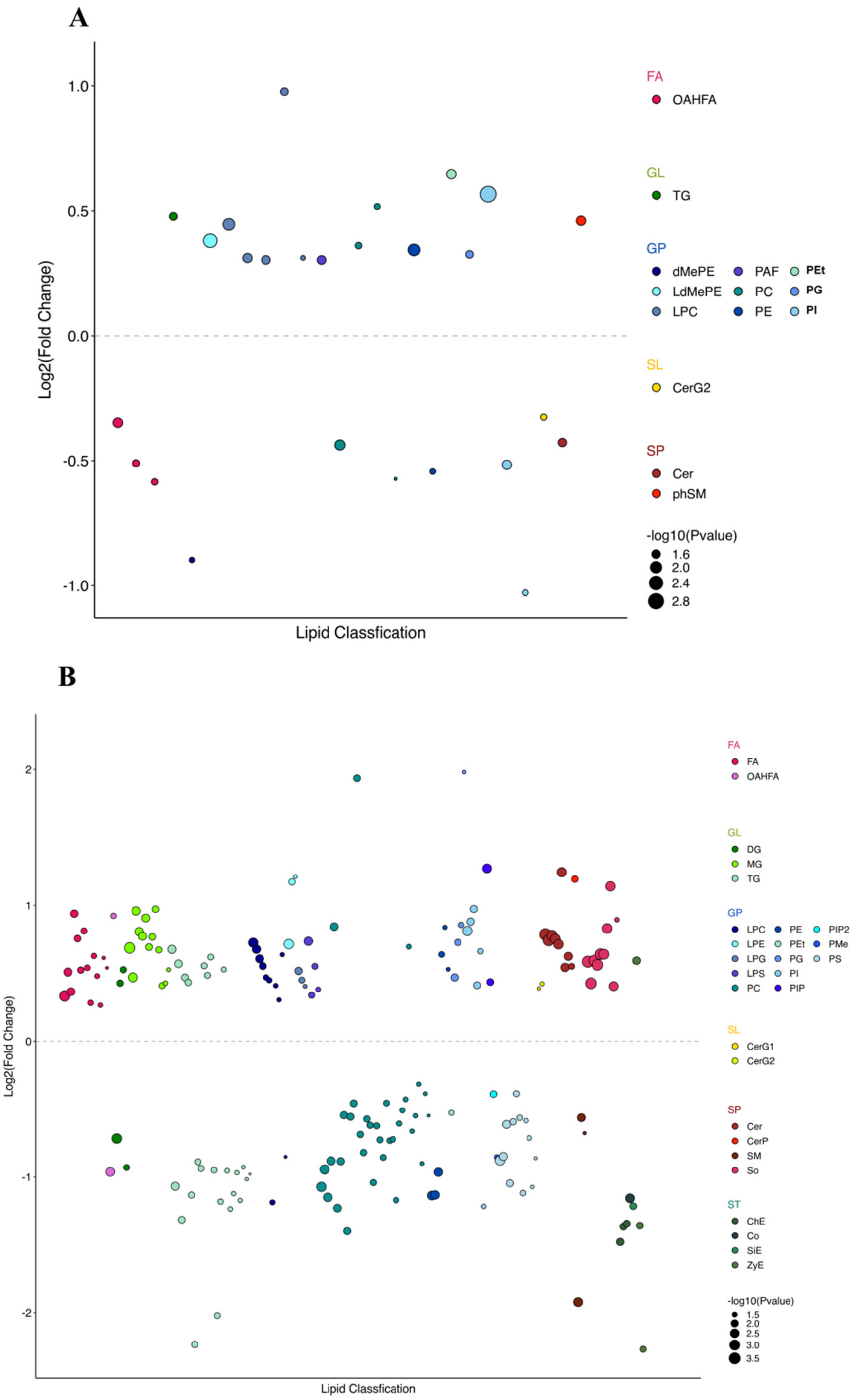

Figure S1. Effect of blue mussel oil on classfication of differential lipid metabolites in plasma of RA rats (n=6). (A) RA group vs Control group, (B) RA+BMO group vs RA group. Differential lipid metabolites were identified based on $p < 0.05$ and $|\text{fold change}| \geq 1.2$. FA: Fatty acyls; GL: Glycerolipids; GP: Glycerophospholipids; SL: Saccharolipids; SP: Sphingolipids; ST: Sterol Lipids.

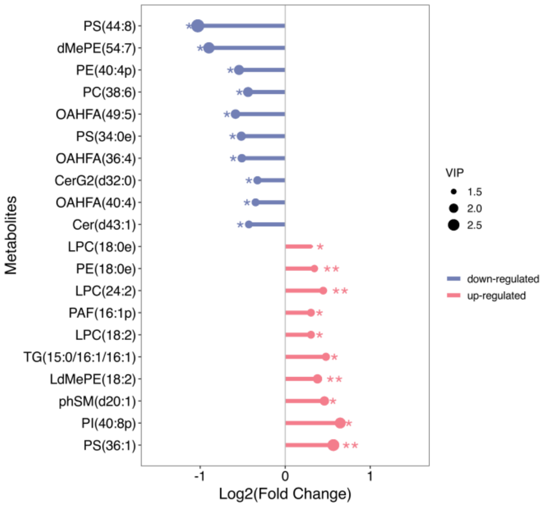

Figure S2. Top 20 significant differential lipid metabolites (RA group vs Control group) in plasma of RA rats (n=6). The top 10 upregulated and top 10 downregulated metabolites with the smallest p values were selected. Red and blue dots indicate upregulated and downregulated metabolites, respectively. *$p < 0.05$; **$p < 0.01$. Dot size reflects the VIP score.

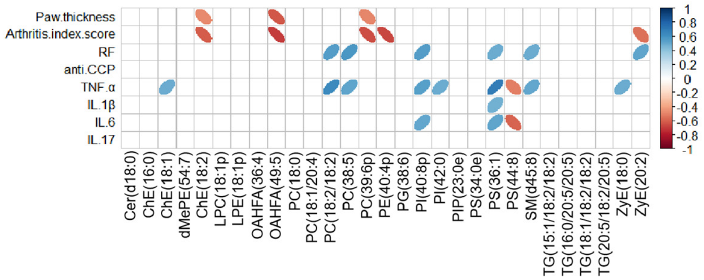

Figure S3. Correlation analysis between significant differential metabolites and RA related indexes (n=6). Correlations were calculated using Spearman's correlation analysis. Blue colors indicate positive correlations; red colors indicate negative correlations. $p < 0.05$ was considered statistically significant.

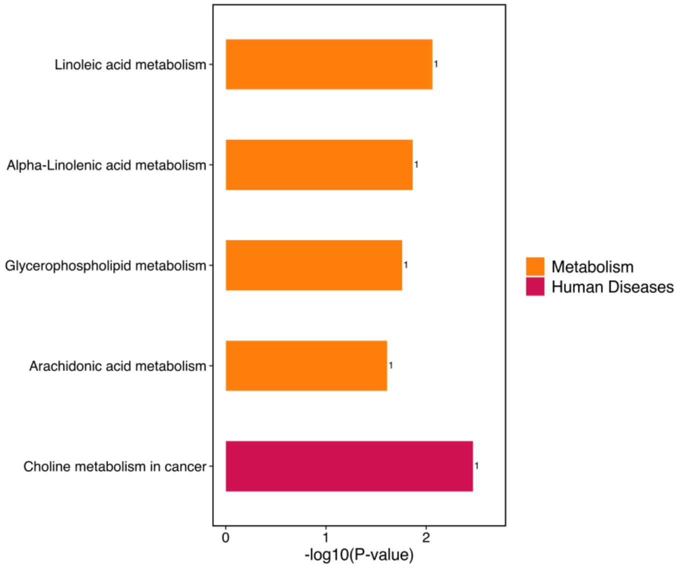

Figure S4. Significant differential KEGG pathways (RA group vs Control group) in plasma of RA rats (n=6). Numbers on the bars indicate the number of differential metabolites mapped to each pathway, and bar colors denote different KEGG Level 1 categories.
